# Identifying the location of a concealed object through unintentional eye movements

**DOI:** 10.3389/fpsyg.2015.00381

**Published:** 2015-04-08

**Authors:** Yair Neuman, Dan Assaf, Navot Israeli

**Affiliations:** ^1^Homeland Security Institute and Department of Education, Ben-Gurion University of the NegevBeer-Sheva, Israel; ^2^Independent ResearcherPetakh-Tikva, Israel; ^3^Wales Ltd.Ramat-Gan, Israel

**Keywords:** cognition, investigation, concealed object, eye movements, endogenous/exogenous attention, Homeland security

## Abstract

In some investigative and interrogative contexts, the investigator is seeking to identify the location of an object (e.g., implanted bomb) which is known to a given subject (e.g., a terrorist). In this paper, we present a non-intrusive methodology for uncovering the loci of a concealed object by analyzing the subject's eye movements. Using a combination of eye tracking, psychological manipulation and a search algorithm, we have performed two experiments. In the first experiment, we have gained 58% hit rate in identifying the location of the concealed object and in the second experiment 56% hit rate. The pros and cons of the methodology for forensic investigation are discussed.

## Introduction

Intelligence and law enforcement agencies are recurrently addressing the challenge of exposing the loci of a concealed object (e.g., bomb), by interrogating people who are suspected of knowing the object's location. For instance, a terrorist is interrogated about the location where he planted a bomb, a woman is investigated about the hiding place of her spouse who is requested for an interrogation and a Hamas terrorist is interrogated about the location of an entrance to an attack tunnel leading from Gaza to Israel.

While traditional interrogation tactics, such as using threat or sleep deprivation, are known to be effective, there are some serious ethical and practical difficulties in using them.

First, in a democratic society it is illegal and unethical to use aggressive investigation tactics on subjects who are not “ticking bombs.” The methodology we present in this paper is nonintrusive and doesn't use aggressive tactics.

Second, conventional interrogation tactics are well-known to terrorists' organizations and therefore suspects may use counter-strategies to mislead the interrogator. Here we present a *novel* methodology which is relatively resilient to the most common counter strategies that a suspect may use.

Third, in such a situation where the interrogation tactic is familiar to the suspect, the traditional interrogation tactics may be time consuming, specifically if there is a pressure of a ticking bomb and the risk to human lives. The novel methodology, we present in the paper involves few minutes, at least in the context where a concealed object is to be identified in a 250 cells matrix.

The aim of the current paper is to introduce a novel methodology for identifying the location of a concealed object through the analysis of the subject's unintentional eye movements.

In this sense, the current project is similar to the those conducted in the context of the *Concealed Information Test* (Ganis and Patnaik, 2009; Ben-Shakhar, 2012; Farwell, 2012; Schwedes and Wentura, 2012) or those conducted in the context of *Lie Detection* (Ganis and Keenan, 2009; Lui and Rosenfeld, 2009; Matsumoto et al., 2011; Rosenfeld et al., 2012; Bowman et al., 2013; Dando et al., 2013; Gamer, 2014), specifically through the use of advanced technologies (e.g., EEG).

The context of lie detection needs no explanation. The concealed information test is used to detect a person's guilty knowledge of a crime. For instance, let us assume that we are investigating a homicide case in which the victim has been choked to death by a string of violin. This knowledge is known only to the murderer and the forensic team. A suspect who is interrogated about the murder is asked: Concerning the weapon used for the murder, was it (1) a knife? (2) a gun? (3) a violin string? (4) a crowbar? (5) a stone? Some physiological measures are taken during the investigation in order to check whether the subjects produces a different physiological response to the item involving the concealed information.

The current project *is different* from the abovementioned research in several aspects. First, we don't aim to differentiate between truth-tellers and liars. Our basic working assumption is that the interrogated subject knows where the object is concealed and will do all necessary efforts, including lying, in order to deceive the investigator.

Second, most of the above-mentioned studies, including those in the Concealed Information tests, suffer from very limited applicability to real-world situations (Ben-Shakhar, 2012) mainly as a result of countermeasures that can be used by the subject and none of them *specifically focuses on the identification of a concealed object*.

In this context, even the use of the most advanced technology is debated (e.g., Farwell, 2012; Meijer et al., 2013). The methodology we present aims to address these difficulties as will be explained below.

## The theoretical rationale

Although the current paper is an instance of applied cognitive science, it is well-grounded in a theoretical rationale as elaborated in this section.

We hypothesized that when presenting the interrogated subject with a visual field where the object is concealed, *unintentional* eye movements may be indicative of the object's location. More specifically, our theoretical rationale is grounded in the context of attention and more specifically on the difference between *endogenous* and *exogenous* attention (Meeter et al., 2010; Mulckhuyse and Theeuwes, 2010) and the idea that our eyes may *deviate from a location where a distractor should appear* (Van der Stigchel and Theeuwes, 2006).

Endogenous attention is a top-down voluntary process while exogenous attention orientation is a bottom-up and stimulus guided process:

“When an observer intentionally selects only those objects required for the task at hand, selection is said to occur in an endogenous, voluntary, goal-directed manner. When specific properties present in the visual field determine selection independent of the observer's goals and beliefs, selection is said to occur in an exogenous, involuntary, stimulus-driven manner” (Meeter et al., 2010, p. 271).

A subject interrogated about the location of a concealed object in a given visual field presented to him, may use very simple counter strategies such as (1) denying that he is familiar with the location of the object, and (2a) moving his eyes randomly or (2b) focusing his attention and gaze on a given loci different from the one in which the object is actually concealed.

However, under a certain interrogative context, exogenous attention orientation may lead to a “slip” of the eyes' fixation *to and away from the concealed object*. In other words, and based on the abovementioned rationale (Van der Stigchel and Theeuwes, 2006), we hypothesized that the location of the concealed object can be inferred from eye movement patterns.

For example, by identifying the locations of the visual scene which the eyes “*avoid*” visiting. Our methodology uses this hypothesis for uncovering the place of the object as instantiated in the algorithm we have designed and applied.

In addition, and as inspired by Milton Erickson's paradoxical approach in hypnosis and psychotherapy, we used a specific paradoxical manipulation in which we have *deliberately instructed our subjects to lie*.

This paradoxical instruction is in line with the findings, cited by Rosenfeld et al., (2012, p. 114) that “forcing participants to give explicitly deceptive responses during the CIT (i.e. Concealed Information Test) will improve its detection efficiency.”

We hypothesized that a paradoxical instruction to lie in a context where the investigator is seeking the truth (or the location of a concealed object) may produce “cognitive load,” that pushes the subjects out of balance, and may help us to better identify “gaze slips” from the location of the concealed object.

## Experiment 1

### Materials and methods

#### Subjects

All of the experiments reported in this paper have been approved by the university ethics committee and informed consent was obtained from the subjects. We recruited subject only with normal eyesight. In Experiment 1, we recruited nineteen female undergraduate university students who voluntarily participated in the study.

#### Apparatus, stimuli, and procedure

We used SMI RED Remote Eye Tracking Device for recording eye movements during the experiments. The system has a sampling rate of 250 Hz and gaze accuracy of 0.4°. The screen resolution was 1680 × 1050 pixels.

The subjects were told that they are participating in an eye movement experiment.

First, the subjects were seated approximately 50 cm from the computer screen and eyes calibration has been performed. Next, a 15 by 15 matrix has been presented on the screen with coordinates appearing at the left column (1–15) and the upper row (A–O).

The subject was asked to chose one of the 225 matrix cells and to write her choice on a paper matrix while the experimenter is unaware of her choice.

Next, she was told that in each step in the experiment, she will be presented with a screen in which a horizontal/vertical line appears, segmenting the matrix into two sections, and that she will be asked whether the cell she has chosen appears above/below to or right/left of the segmenting line. The subject was instructed to deliberately try and deceive the experimenter.

Each experiment consisted of 28 trials where the segmenting line coordinates have been randomly generated and no time limits were imposed on the subject's response time. The experiment moved to the next screen whenever an answer was given by the subject.

This decision making procedure, under the paradoxical instruction to deceive the experimenter, aimed to expose the exogenous attention orientation as “running away” from the chosen location.

The SMI eye tracking device was used to record the subjects' eye fixations during each trial and the data were used for the analysis and the identification of the cell the subject has chosen.

Two points must be clarified.

First, each participant choose only one *target location* and each experiment represented multiple attempts to infer the location of that single location.

Second, it must be noted that in order to deceive the experimenter the subject could have stick to a single answer but could have also used a mix strategy of lying and telling the truth, which is precisely the strategies used by the subjects. In most of the cases, the subjects chose to stick to a single answer only. Therefore, the subject's answers have no informative value for identifying the location of the concealed object.

The reason we have asked the subjects to provide verbal answers is the same reason we have asked them to deceive the experimenter or used the line in segmenting the matrix; The attempt to increase cognitive load and to remove the subject's conscious control of his/her eye movements.

#### The algorithm

We have designed a unique algorithm that aims to model the subject's eye movements for identifying the location of the concealed object.

The algorithm is not the simple result of “engineering” efforts *per se* but corresponds with the theoretical rationale as presented above. In a nutshell, and following the attention-based hypothesis presented above, the algorithm we have designed ranks the probability that the target is located in a given cell, based on the entropy calculation of *shifting* the eye's fixation from a specific cell to other cells in the matrix. Let us explain the algorithm.

We attempt to discover the target cell for each experiment (= subject) using the eye data collected during all the trials. The output of an experiment includes a list of events of the following types:

1. Fixations – characterized by start and end times (in Milliseconds), average eye location [in pixels (1680 × 1050)], eye location dispersion (in pixels), and average pupil size.
2. Saccades – start and end times (in Milliseconds), start and end locations (pixels), speed and acceleration measures.
3. Blinks – start and end time (Milliseconds).

These events are analyzed by the experimental apparatus (SMI RED Remote Eye Tracking Device).

In this study, we analyze only fixation events and use only the start and end times and start and end locations. Thus, the input to our algorithm is a series of eye fixations.

We sample this series with a 0.1 s (~average fixation time) and 105 × 65 pixels (cell size) resolution to get a trajectory: eye location (in cells) as a function of time.

For the eye trajectory, we calculate a cell transition matrix assuming the trajectory was generated by a Markov process. That is, we analyze the process in which the fixation is moving from one cell of the matrix presented to the subject to another cell of the matrix presented to the subject.

We use the transition matrix to calculate the process entropy production and the entropy production of individual cells. Our motivation for measuring entropy is as follows:

1. Our goal is to find the matrix's cell that best “explains” the subjects eye movements.
2. Information theoretically, we can interpret this as looking for a context (i.e., the target cell) that minimizes the information content of the eye trajectory. This information content is called the “Kolmogorov Complexity” or “Algorithmic Complexity” of the trajectory.
3. Kolmogorov Complexity of data is incomputable. However, for Markov processes, the process entropy production asymptotically approaches the Kolmogorov complexity of the process output. This is our reason for modeling the data as a Markov process and for measuring the process entropy production.
4. We selected to work with 0.1 s time resolution because this is the average fixation time. In this way, equal weights are given to Markov transition between cells and Markov transitions within the same cells. We did check other sample times and our results are very robust with regard to this parameter.

Now let us present the algorithm for identifying the location of the subject's target.

As mentioned above, we start with high resolution (pixel size, milliseconds) eye fixation measurements. The data includes fixation coordinates, start and end time as well as other parameters. The high resolution data is sampled into a trajectory with cell size and ~0.1 s resolution.

Let (*i*_*t*_, *j*_*t*_) be the eye cell positions in an *N* × *N* grid for time steps *t* = 1 … *T*.

We assume that this trajectory is a result of a Markov process and calculate the process transition probabilities *P*_*ij, kl*_ from cell *ij* to cell *kl* as follows:

1. Set *P*_*ij, kl*_ = 0 for all *ij*, *kl*.
2. Count transitions:
  ◦ For *t* = 1 … *T* − 1
    ▪ *P*_*i*_*t*_*j*_*t*_,*i*_*t*+1_*j*_*t*+1__ = *P*_*i*_*t*_*j*_*t*_,*i*_*t*+1_*j*_*t*+1__ + 1
  ◦ End
3. Normalize:
  ◦ For *i* = 1..*N*, *j* = 1..*N*
    ▪ *Z* = ∑_*kl*_ *P*_*ij,kl*_
    ▪ For *k* = 1..*N*, *l* = 1..*N*
      - *P*_*ij,kl*_ = $\frac{P_{ij,kl}}{Z}$
    ▪ End
  ◦ End
4. Calculate cell entropy:
  ◦ For *i* = 1..*N*, *j* = 1..*N*
    ▪ *H*_*ij*_ = −∑_*kl*_ *P*_*ij,kl*_ · *logP*_*ij,kl*_ (using the convention 0*log*0 = 0)
  ◦ End

Entropy matrices for different tests were calculated and we observed that target cells were well-correlated with:

1. Entropy value – target cells tend to have high entropy
2. Spot size – target cells entropy peaks are narrow

To include these two observations in a selection method, we first identified clusters/spots on the entropy map. Starting with a cell at a local peak, we clustered together all its neighbors (recursive) whose entropy values exceeded a relative threshold (0.25). This was done for a few (3) dominant peaks. All the cells that were left un-clustered by this process were then grouped together as a background cluster.

Using the entropy measure and the clustering, we finally calculated a selection score *S*_*ij*_ = *H*_*ij*_ · *exp*(−α · *C*_*ij*_) where *C*_*ij*_ is the size of the cluster cell *ij* belongs to. The selected cell is actually the one that gains the MAX selection score.

In sum, the algorithm actually measures the transition scores for cells by counting “out degree” trajectories of eye fixation and by operating a decision rule as described above. It must be noted that the eye-tracking analysis was done offline after the experiment was over and not for each step or in real time.

**Figure 1:**
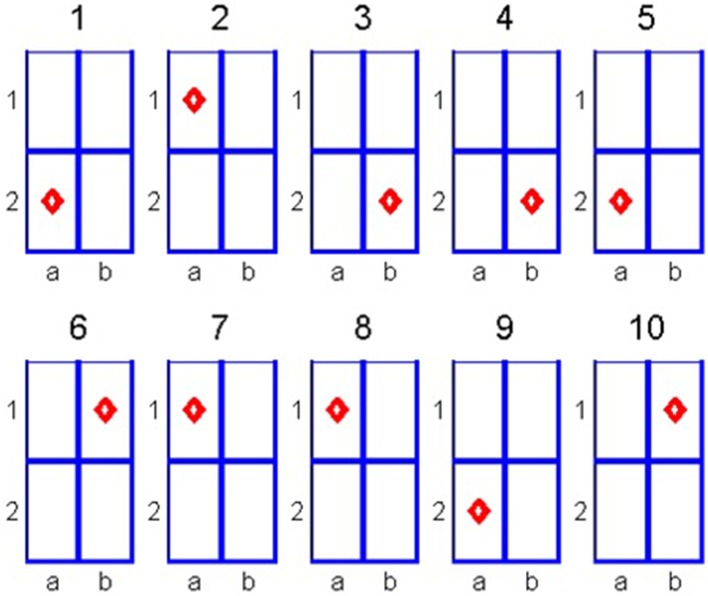
**Eye fixations for the 2 × 2 matrix**.

Let us illustrate the algorithm through a simple example. Suppose that we have a 2 × 2 cell matrix. The subject is asked to choose one of the cells and we aim to identify the location of the chosen cell by analyzing the subject's eye fixations. In our example, we analyze 10 eye fixations as depicted in Figure 1.

Note that in steps 4 and 5 the fixation is on the same cell. This can be the result of two consecutive fixations on the same cell or a single long fixation that exceeded the used trajectory sampling time. We count the following transitions:

**Table:**
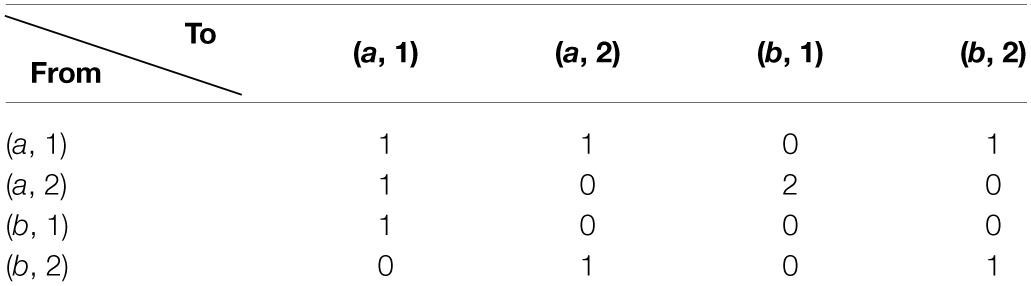


Modeling this as a Markov process we have the transition matrix

$P=\left(\begin{array}{cccc} \frac{1}{3} & \frac{1}{3} & 0 & \frac{1}{3}\\ \frac{1}{3} & 0 & \frac{2}{3} & 0\\ 1 & 0 & 0 & 0\\ 0 & \frac{1}{2} & 0 & \frac{1}{2} \end{array}\right).$ The entropies of the different cells are the sum of −*p* · *log*(*p*) over the rows of the transition matrix. The values are:

$$\begin{array}{l} H_{a,1}=-3*\frac{1}{3}\log\left(\frac{1}{3}\right)\approx 1.1\\ H_{a,2}=-\frac{1}{3}\log\left(\frac{1}{3}\right)-\frac{2}{3}\text{log}\left(\frac{2}{3}\right)\approx 0.64\\ H_{b,1}=-\log\left(\text{1}\right)=0\\ H_{b,2}=-2*\frac{1}{2}\log\left(\frac{1}{2}\right)\approx 0.69. \end{array}$$

**Figure 2:**
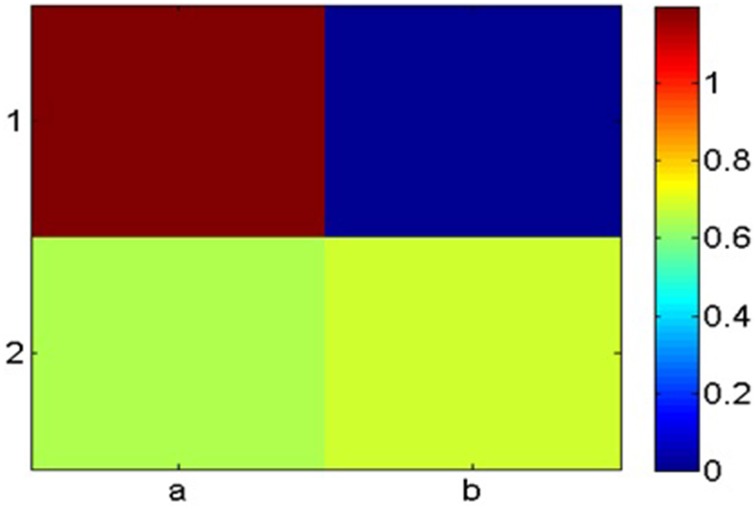
**Heat map of the matrix according to entropy values**.

Coloring the cells according to their entropy values, we obtain the map presented in Figure 2.

Analyzing this map, we identify the peak value which is (*a*, 1) in the above example. As we previously explained, peak width was also found to be an effective feature for guessing target cells. This feature however is meaningful only when the peak width is much smaller than the matrix size. Our 2 × 2 example is not instructive in this respect and used for general illustration only.

### Results

Given the fact that the eye fixation is imbued with noise and that the measurement is noisy, we considered a successful identification of the chosen location if the algorithm identified the exact cell chosen by the subject or one of its neighboring cells.

The algorithm successfully identified the location of the cell chosen by the subject in 11 out of 19 cases, which is 58% hit rate. In 6 out of 11 (55%) cases the algorithm identified the exact location of the cell and in the other cases, it missed the target by one cell only.

As the matrix presented to the subjects includes 225 cells, the chance of randomly hitting the target is 0.004, and 0.04 if we allow a deviation of one cell from the subject's chosen cell. Therefore, by using the Binomial test we can easily verify that the algorithm hit rate is a statistically significant improvement over a random guess (*p* < 0.001).

**Figure 3:**
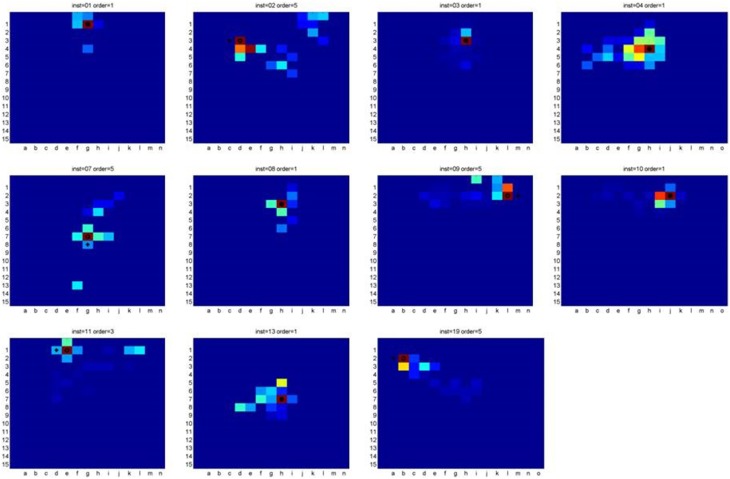
**Heat maps of the cases where the algorithm successfully identified the chosen cell**.

Figure 3 presents the heat-maps of the cases where the algorithm identified the concealed object's location. The cross marks the target cell while the circle marks the algorithm's guess.

Based on the hypothesis that the eye's first fixations on the first screen presented to the subject may be indicative of the object's location, we have used a simple heuristics to identify the target cell in cases where our algorithm failed to identify it. It must be noted that *when used alone this heuristics fails* to generate any significant results and it can be used only as a practical complementary procedure for the algorithm presented above.

We identified the first three fixations and searched for the target in a serial process starting with the first fixation, moving to the neighboring cells, to the cell's second fixation and so on.

Using this heuristics, we were able to identify the target cells chosen by two additional subjects, and increased the hit rate to 68% by “paying” the price of searching 25 additional cells on average (11%) for each subject.

The first experiment involved a selective group of female university students who didn't have a significant incentive to successfully deceive the experimenter. Experiment 2 aimed to address these difficulties.

## Experiment 2

### Subjects

The second experiment involved 18 subjects (89% male) from various ethnic background, education levels, and age (range 20–66). The subjects were recruited from a local locksmith's workshop in a Southern Israeli city, from several customers who arrived to the place and from blue collar workers of nearby workshops.

### Procedure

We used a variation of the procedure applied in Experiment 1.

First, we used different instructions to address the specific audience. The subjects were presented with the matrix and were told that the matrix represents floor tiles under which one of them they should hide money. They were asked to sketch the place where they hide the money on a paper representation of the matrix without the experimenter's knowledge.

Next, they were told that at each trial the matrix will be presented on the screen with a line segmenting the matrix and that they will be asked whether the location of the hidden money is above/below or to the right/left of the line.

They were also told that they should deceive the experimenter by lying to him and that a successful deception will be compensated by 50 NIS (~$13). Regardless of the subject's true success s(he) was compensated for the participation. The sum paid to the subjects for a 10 min experiment is approximately twice the minimum wage salary per hour and therefore it is a significant incentive for most of the participants to successfully deceive the experimenter.

In addition, the exposure time for this experiment was set to 1.5 s and the subject was exposed to 18 trails in which the line randomly segmented the screen.

### Results

The algorithm identified the exact location of the target or deviated in a single neighboring cell from the target cell, in 10 out of 18 cases which mean 56% hit rate. Applying the heuristics described in Experiment 1 on the 8 other cases, we have identified three additional targets, gaining an overall hit rate of 73%, at the price of searching on average additional 18 cells (8%) for each subject.

### Discussion

The current paper presents a novel, theoretically grounded and empirically proven methodology for the identification of a concealed object based on the analysis of the subjects' exogenous attention through his eye movements.

The results present a significant improvement over a random search by using a non-intrusive methodology/tactic of investigation. As our methodology is novel, it could have been compared to a random search only. There is only one similar attempt to identify the location of a concealed object through eye movements analysis. This methodology that analyzes micro saccades, is reported in a patent (Martinez-Conde et al., 2010) but there is no published scientific evidence regarding its efficiency. When applying the proposed methodology by Martinez-Conde et al. (2010) using the analysis of saccades, no significant results were gained in identifying the location of a concealed object in our data.

While all of the subjects realized that their eyes are monitored, some of the subjects, successfully used a simple counter strategy in which they have intentionally focused their eyes on a location different from the one they have originally chose. Addressing this counter strategy through the fusion of other information sources and biological signals such as GSR, may improve our results. That is, an improvement in the methodology's performance should include a better procedure that addresses the counter-strategy and the fusion of several physiological signals.

In real world investigation, the subject might refuse to cooperate and look at the screen. However, this move turns him into a suspect that has something to hide and in this respect it is a self-defeating and incriminating strategy.

In sum, the current paper presents a preliminary and basic methodology that can be used and attuned to a wide variety of forensic investigative contexts in which the loci of a concealed object has to be identified through the investigation of a human subject. In comparison with other technologies such as EEG and fMRI, the methodology is relatively cheap, produces robust results in short time and cannot be easily resisted. The practical applications of this methodology should be further developed in order to establish it as a working tool for law enforcement agencies.

### Conflict of interest statement

The authors declare that the research was conducted in the absence of any commercial or financial relationships that could be construed as a potential conflict of interest.
